# Development of a genome‐wide 200K SNP array and its application for high‐density genetic mapping and origin analysis of *Camellia sinensis*

**DOI:** 10.1111/pbi.13761

**Published:** 2021-12-24

**Authors:** Kang Wei, Xinchao Wang, Xinyuan Hao, Yinhong Qian, Xin Li, Liyi Xu, Li Ruan, Yongxin Wang, Yazhen Zhang, Peixian Bai, Qiang Li, Shirin Aktar, Xili Hu, Guoyang Zheng, Liubin Wang, Benying Liu, Weizhong He, Hao Cheng, Liyuan Wang

**Affiliations:** ^1^ Key Laboratory of Tea Biology and Resources Utilization Ministry of Agriculture National Center for Tea Improvement Tea Research Institute Chinese Academy of Agricultural Sciences (TRICAAS) Hangzhou China; ^2^ Lishui Academy of Agricultural Sciences Lishui China; ^3^ Tea Research Institute Yunnan Academy of Agricultural Sciences Menghai China; ^4^ Jinyun Agricultrual Bureau Jinyun China

**Keywords:** *Camellia sinensis*, SNP array, seed setting rate, genetic map, demonstration

Tea is one of the most popular drinks in the world. It is a self‐incompatible plant species, and its self‐incompatibility greatly contributes to its high genetic diversity. Cultivated tea plants mainly include *Camellia sinensis* var. *assamica* (CSA) and *Camellia sinensis* var. *sinensis* (CSS). The former is characterized by large leaves, cold sensitive phenotype, and an arborous or semi‐arborous habit, whereas CSS is characterized by smaller leaves, cold tolerance, and a shrub or semi‐shrub growth habit. However, their genetic diversity and differentiation, especially the original relationship, still remain elusive.

Earlier publications revealed a total of 218.9 million high‐quality variants based on the chromosome‐level reference genomes and re‐sequencing data of tea plants (Wang *et al*., 2020). Following the SNP selection process, 5 360 472 SNPs were selected with minor allele frequencies >0.1. Then considering the genome‐wide uniform distribution and location of SNPs on or close to genes, a set of 179 970 SNPs uniformly distributed across the genome were chosen for the development of the 200K Affymetrix Axiom SNP array. Relative information was deposited in the National Center for Biotechnology Information with accession number of GSE182082 (https://www.ncbi.nlm.nih.gov/geo/query/acc.cgi?acc=GSE182082). All samples passed the quality assessment with an average call rate of 98.1%. The SNP reproducibility of duplicated samples was 99.71% for ‘LJ43’ and 99.67% for ‘BHZ’. The consistent rates of the genotyped SNPs of the SNP array and re‐sequencing data of 21 overlapped tea cultivars ranged from 64.14% to 91.93%, with an average of 84.07%. We further analyzed the discrepancy types of the genotyped SNPs in the overlapped cultivars (Figure 1a). Most of the discrepancies (78.3%) occurred as heterozygous by re‐sequencing, but homozygous by the SNP array. We then randomly selected 18 discrepant SNPs and performed Sanger sequencing PCR for verification. Eight of them were consistent with the SNP array results, and ten were consistent with the re‐sequencing data. It was found that re‐sequencing tends to overestimate heterozygous sites, while the SNP array tends to mistake heterozygous sites for homozygous sites. Torkamaneh *et al*. (2016) found that SNP catalogues called from different pipelines or technologies might not be consistent. Here, our results showed that neither re‐sequencing nor the SNP array can guarantee 100% correct results. Therefore, the mutual verification of different methods is very important.

**Figure 1:**
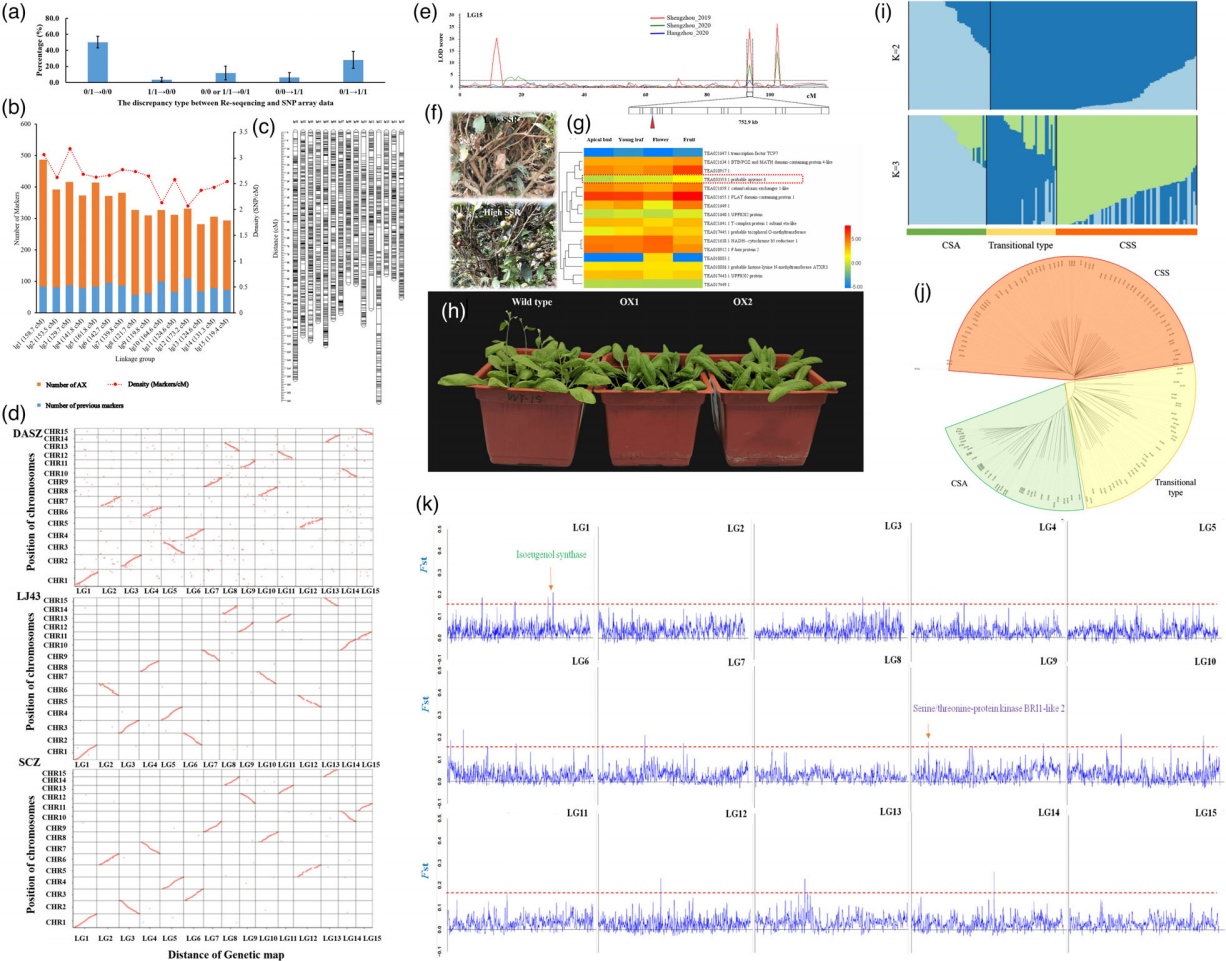
Genetic distribution and application of the Tea 200K array SNPs. (a) Percentage of each discrepancy type between re‐sequencing and SNP array data. 0 and 1 represent reference and alternative base, respectively. (b) Genome distribution of the bin markers in the 15 linkage groups. (c) High‐density genetic map. (d) Collinearity of the genetic map and three published tea genomes. (e) Major QTL of SSR in LG15. (f) Phenotype of high and low SSR tea plants. (g) Tissue expressions of 16 candidate genes. (h) Phenotypes of the *Arabidopsis* transgenic lines of *CsAPY6* (OX1 and OX2) and wild‐type. (i, j) Structure and phylogenetic analysis of 142 tea cultivars. (k) Distribution of windowed *F*_ST_ values along each chromosome between CSS and the remaining tea cultivars.

To construct a high‐density genetic map, an F1 population with 327 individuals was genotyped by the tea 200K SNP array. After filtering for quality and missing data, a total of 18226 SNPs were found to be polymorphic. All these SNPs were merged into the existing genetic linkage map using 2b‐RAD sequencing (Xu *et al*., 2018), leading to about three‐fold increase in the markers. After binning of redundant markers, totally 5325 bin markers over 15 linkage groups (LGs) covering 2107.01 cm were obtained (Figure 1b,c). The distances of the bin markers ranged from 0.31 cm in LG3 to 0.52 cm in LG12, with an average of 0.39 cm.

We then performed chromosomal collinearity analysis of the high‐density genetic map and the published CSS (‘SCZ’ and ‘LJ43’) and ancient tea (‘DASZ’) genomes (Figure 1d). The genetic map is highly consistent with the published tea genomes, but better collinearities were identified with the CSS than those of ancient tea, suggesting the existence of a lot of differences in each chromosome of CSS and ancient tea.

To validate the application of the high‐density genetic map, we used the F1 population to measure a trait ‘seed setting rate (SSR)’ (Figure 1e,f). The phenotypic data were collected from the population grown at Hangzhou in 2020 and Shengzhou from 2019 to 2020, China. A total of four overlapped QTLs were identified, which were mapped to LG1, 2, 5 and 15, respectively. Taking the QTL in LG15 for example, it was then mapped to the reference tea genome of ‘SCZ’ (Xia *et al*., 2020), with an interval of 752.9 kb. A total of 16 genes were identified in this region. Among them was an apyrase gene (TEA033353.1) showing higher expressions in its flower and fruit (Figure 1g). Its homologous genes in *Arabidopsis* (*AtAPY6* and *AtAPY7*) are closely associated with fertility (Yang *et al*., 2013). To verify the function of TEA033353.1, it was expressed under the control of the CaMV *35S* promoter in *Arabidopsis* (Figure 1h). Surprisingly, the transgenic lines showed late bolting and flowering phenotypes, indicating that it negatively controls fertility. This case study also suggests that the SNP array is useful for further genetics research.

To gain a better understanding of the domestication and classification of tea, we performed population structure and phylogenetic analysis of 142 tea cultivars bred from different provinces of China (Figure 1i–k). At a K value = 3, CSA, CSS, and a transitional type could be clearly distinguished (Figure 1i). Many cultivars in the transitional type were artificial hybrids from CSA and CSS, indicating that they are the transition between the two types. The phylogenetic tree analysis is consistent with the structure results (Figure 1j). Moreover, we found that cultivars of CSS were clustered tightly together in the phylogenetic tree, suggesting that the genetic variation within the CSS group was much less than that among the remaining cultivars. The position of CSA, CSS, and the transitional type reflects their evolutionary relationship.

We then calculated Wright’s *F*‐statistic (*F*_ST_) to estimate the genetic divergence of CSS and the remaining cultivars (Figure 1k). There were distinct patterns in the distribution of windowed *F*_ST_ values for each LG. A total of 1.24% of the *F*_ST_ windows were under strong selective sweeps (*F*_ST_ > 0.15), with large effects mainly in LG1, 9 and 13. Interestingly, we identified two selection signatures in the CSS population. One is an isoeugenol synthase gene cluster in LG1 correlated with the biosynthesis of (E)‐isoeugenol, a characteristic aroma component of green tea (Baba and Kumazawa, 2014). The other is a BRI1‐like 2 gene (TEA021769) in LG9, whose expression in CSA is >10‐fold higher than that in CSS. BRI1 is a receptor kinase of brassinosteroids controlling plant development (Tang *et al*., 2008). Further exploitation of these genes would contribute to the interpretation of the CSS evolution process.

In summary, our SNP array data show that it is a useful platform for original analysis and forward genetics research.

## Conflict of interest

The authors declare no competing financial interests.

## Author contributions

K.W., X.W., X.H., Y.Q., and X.L. carried out data analysis and drafted the manuscript. L.X. constructed the genetic linkage map. L.R., Y.W., Y.Z., P.B., Q.L., S.A., S.X., X.H., G.Z., B.L., M.H., and L.W. collected samples and performed the experiments. X.W. and W.H. discussed the results. H.C. and L.W. managed the research.
